# TSC loss distorts DNA replication programme and sensitises cells to genotoxic stress

**DOI:** 10.18632/oncotarget.13378

**Published:** 2016-11-16

**Authors:** Govind M. Pai, Alexandra Zielinski, Dennis Koalick, Kristin Ludwig, Zhao-Qi Wang, Kerstin Borgmann, Helmut Pospiech, Ignacio Rubio

**Affiliations:** ^1^ Institute of Molecular Cell Biology, Center for Molecular Biomedicine, University Hospital, 07745 Jena, Germany; ^2^ Laboratory of Radiobiology & Experimental Radiooncology, Department of Radiotherapy and Radiooncology, Center of Oncology, University Medical Center Hamburg-Eppendorf, Germany, 20246 Hamburg, Germany; ^3^ Leibniz Institute on Aging - Fritz Lipmann Institute, 07745 Jena, Germany; ^4^ Faculty of Biochemistry and Molecular Medicine, University of Oulu, 90014 Oulu, Finland

**Keywords:** tuberous sclerosis, mTORC1, replication stress, genotoxic stress, adaptive responses

## Abstract

Tuberous Sclerosis (TSC) is characterized by exorbitant mTORC1 signalling and manifests as non-malignant, apoptosis-prone neoplasia. Previous reports have shown that TSC^-/-^ cells are highly susceptible to mild, innocuous doses of genotoxic stress, which drive TSC^-/-^ cells into apoptotic death. It has been argued that this hypersensitivity to stress derives from a metabolic/energetic shortfall in TSC^-/-^ cells, but how metabolic dysregulation affects the DNA damage response and cell cycle alterations in TSC^-/-^ cells exposed to genotoxic stress is not understood. We report here the occurrence of futile checkpoint responses and an unusual type of replicative stress (RS) in TSC1^-/-^ fibroblasts exposed to low-dose genotoxins. This RS is characterized by elevated nucleotide incorporation rates despite only modest origin over-firing. Strikingly, an increased propensity for asymmetric fork progression and profuse chromosomal aberrations upon mild DNA damage confirmed that TSC loss indeed proved detrimental to stress adaptation. We conclude that low stress tolerance of TSC-/- cells manifests at the level of DNA replication control, imposing strong negative selection on genomic instability that could in turn detain TSC-mutant tumours benign.

## INTRODUCTION

Eukaryotic cells coordinate cellular growth with nutrient availability and environmental favourability. A highly conserved member of the atypical Phosphoinositide 3-kinase-related kinase (PIKK) family of Ser/Thr kinases, mTOR (mammalian or mechanistic target of Rapamycin), has emerged as a critical signalling hub, functioning as an integrator of diverse inputs regulating cell size, metabolism and growth upon receiving hormonal stimulatory signals via Ras and PI3K (Phosphoinositide-3-kinase) [1-3]. Best studied for its role in translation master-control, mTORC1 (mTOR Complex1) integrates a variety of extraneous and intrinsic stimuli viz., hormones (insulin) and nutrient (amino acids) availability to fuel cell growth by directly controlling protein synthesis and further anabolic and catabolic processes [4]. The tumour suppressor complex TSC1-TSC2 (Hamartin–Tuberin, collectively TSC) is a signalling nexus that negatively regulates mTORC1 activity by functioning as a GTPase-activating protein for the small GTPase Ras homologue enriched in brain (Rheb) [5-7]. Many environmental and intracellular cues that impinge on mTORC1 funnel through TSC and regulate its activity towards Rheb [8, 9]. Tuberous Sclerosis (TS) results from mTORC1 dysinactivation upon mutational loss of TSC1/2 and is characterised by multiple benign hamartomatous tumours in the brain, skin, heart, kidneys and lungs [10, 11]. However, in contrast to other tumours driven by upstream mutations affecting the very same pathway, loss of TSC function manifests as highly apoptotic non-malignant neoplasia suggesting that exorbitantly high mTOR activity in TSC^-/-^ cells impedes full blown tumour development.

Several reports have shown that TSC^-/-^ cells are not only maintained in a benign state but also susceptible to sub-lethal genotoxic stress, at least in part by increased p53 stabilisation and function [12, 13]. Studies thereafter unveiled energetic shortfall and a metabolic collapse due to increased anabolic demand and a lack of resource-sensing as further sensitizing cues in TSC^-/-^ cells [14]. Beyond its well-established role as metabolic master switch, mTORC1 gained attention as a cell cycle regulator after its discovery as Rapamycin's (Rapa) cellular target [15] mediating the block in T-cell proliferation [16]. mTOR's critical function in cell cycle progression was thereafter extended to several tumour cell types [17, 18]. In addition to identifying the obligate requirement of S6K activity for G1–S transition [19, 20], much work has focused on the control of expression of Cyclins D, E, A, and the cyclin-dependent kinase (Cdk) inhibitor p27 by mTORC1 [21]. Accordingly, imbalances in G1/S phase Cyclins or p27 expression [22] along with further cell cycle disturbances like stunted G1 phase and a prolonged S phase [23] have been described as characteristics of cells with constitutive mTORC1 activity. Although much less studied, recent work suggests mTORC1 plays a role in controlling mitotic entry of cells by modulating Cdk1 activity. mTORC1 signalling, therefore, appears to control both G1–S transition and mitotic entry in eukaryotes [24]. Together, high mTORC1 activity by way of protein abundance and increased cell mass, along with high G1 Cdk activity appears to shorten the length of G1 phase and drive premature S phase entry.

Here we address the nature of these deleterious cell cycle alterations in TSC^-/-^ cells and whether this has implications for their proven sensitivity to genotoxic stress. We document that TSC loss, probably as a result of exorbitant mTORC1 activity, predisposes tumour cells to otherwise harmless doses of genotoxic stress by disturbing the cellular DNA replication programme. Based on our findings, we discuss issues inherent to tuberous sclerosis, also reasoning why TSC tumours remain benign by the very virtue of their genotypic constitution.

## RESULTS

### TSC1 loss predisposes cells to genetic damage and cell death

**Figure 1:**
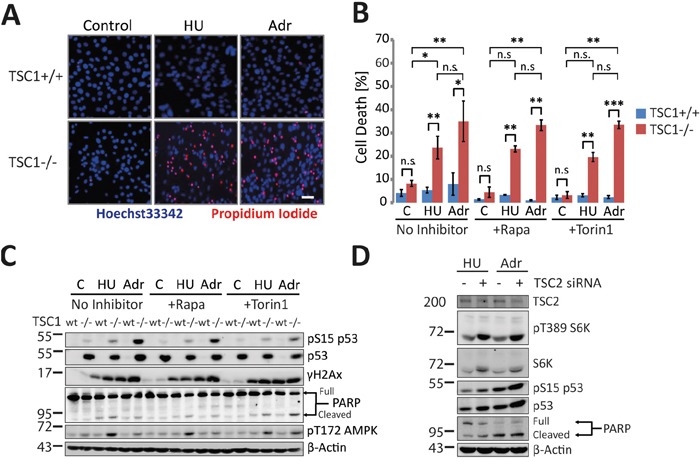
TSC1 loss predisposes cells to genotoxic stress-induced cell death **A.** Fluorescent microscopy of TSC1^+/+^ and TSC1^-/-^ MEFs untreated or treated with Hydroxyurea (HU, 2 mM) and Adriamycin (Adr, 0.5 μg/ml), respectively for 20 h. Hoechst33342 (membrane permeable, live-cell nuclear stain) and Propidium iodide (membrane impermeable dead cell stain) mark live and dead cells. Scale bar = 20 μm. See also Supplementary Figure S1. **B.** Propidium iodide exclusion flow cytometry for cell death estimation. TSC1^+/+^ and TSC1^-/-^ MEFs untreated or treated with Hydroxyurea (HU, 2 mM) and Adriamycin (Adr, 0.5 μg/ml) respectively for 20h. Wherever indicated, mTORC1 Inhibitors Rapamycin (Rapa, 20 nM) or Torin1 (10 nM) were spiked 2h prior to genotoxic treatments. Data-set are a mean of duplicate samples from three independent experiments. Error bars represent standard deviation (SD) of the three repeats. One-way non-parametric ANOVA (for group comparisons) and the non-parametric Mann-Whitney U test (for pair-wise comparison) were used for statistical analysis. *P<0.05, **p<0.01, ***p<0.001, n.s.- not significant. Pair-wise significance is as indicated (TSC1^+/+^ vs TSC1-/-). See also Supplementary Figure S5. **C.** Representative western blot of WT and TSC1^-/-^ MEFs treated as in (B). **D.** siRNA-mediated acute knockdown of TSC2 in wild type MEFs followed by genotoxic treatment and immunoblot detection of the indicated proteins.

In line with previous reports [12], we observed that acute exposure of TSC1^-/-^ MEFs to subtle doses of the genotoxic agents hydroxyurea (HU, a ribonucleotide reductase inhibitor) and adriamycin (Adr; a topoisomerase II inhibitor causing indirectly DNA double strand breaks) resulted in precipitous detachment and cell rounding indicating loss of cell viability (Supplementary Figure S1), whereas both treatments were well tolerated by their wild-type TSC1^+/+^ counterparts. Cell death was significantly higher in TSC1^-/-^ MEFs subjected to low-level DNA damage, as confirmed by assessing plasma membrane permeability to propidium iodide (Figure 1A, 1B). In accordance, TSC1^-/-^ MEFs generally accumulated and stabilised p53 and showed elevated levels of phosphorylation of p53 at serine15 upon genotoxic insult (Figure 1C) [12]. Higher basal as well as stress-induced p53 protein and phosphorylation levels have been reported before for TSC null cells [12, 14]. However, the role of p53 in cell death induced by various types of insult varied strongly in dependency of the particular type of stress applied [25, 26]. To test if p53 was involved in the cellular death pathways evoked by DNA damage in our experiments we recapitulated the findings in TSC2^-/-^ and p53^-/-^ double knockout MEF cells (Supplementary Figure S2). Although TSC2^-/-^ p53^-/-^ and isogenic p53^-/-^ MEF cells accumulated comparable levels of DNA damage in response to a sub-lethal dose of Adriamycin, TSC2^-/-^ p53^-/-^ cells exhibited a stronger increase in cell death compared to p53^-/-^ cells that display intermediate death levels between wt and the two TSC negative lines (Figure 1B, Supplementary Figure S2). Beyond corroborating the higher stress sensitivity of cells with functional TSC loss, these data indicated that p53 was not necessary for death induction under these conditions. Acute knockdown of p53 using siRNA in TSC1^-/-^ confirmed this notion (Supplementary Figure S3). Thus, we conclude that TSC null cells were extremely sensitive to low-dose genotoxic stress but high p53 levels were neither responsible for this sensitization nor required for the cell death phenotype.

To check if TSC^-/-^ cells featured an exaggerated immediate response to DNA damage we performed immunoblot analysis of classical DNA damage response pathways. S15-phosphorylated p53, cleaved Poly-ADP Ribose Polymerase (PARP) (Figure 1C) and cleaved Caspase 3 (Supplementary Figure S4) were all markedly elevated in TSC^-/-^ MEFs compared to their wild-type counterpart MEF cells indicating a low threshold of damage tolerance in those cells. Interestingly, further measurements performed with the intention to characterise cell death mechanisms, yielded only a minor fraction of Annexin-V positive apoptotic TSC1^-/-^ cells under these conditions (Supplementary Figure S5). To understand if the hypersensitivity of TSC^-/-^ cells to stress was reversible we used pharmacological approaches to inhibit mTORC1. Acute allosteric (Rapamycin) or ATP-competitive (Torin1) mTORC1 inhibition, despite reducing total p53 levels and p53 phosphorylation moderately in TSC^-/-^ cells (Figure 1C), conferred little or no protection from cell death (Figure 1B), corroborating that mechanisms other than p53 accumulation rendered TSC-deficient cells hypersensitive to DNA damage. Moreover, the inefficacy of acute mTORC1 inhibition indicated that long-term effects of mTORC1, like e.g. translational effects, probably mediated the increased sensitivity to DNA damage. Upon verifying whether all these observations were a sole consequence of TSC1 or TSC2 ablation and not confounded by high rates of mutations in culture resulting in mal-adaptations, we ascertained that an acute siRNA-mediated knockdown of TSC2 essentially recapitulated salient features of TSC1^-/-^ genotype with regard to their signalling pattern and stress sensitivity (Figure 1D).

**Figure 2:**
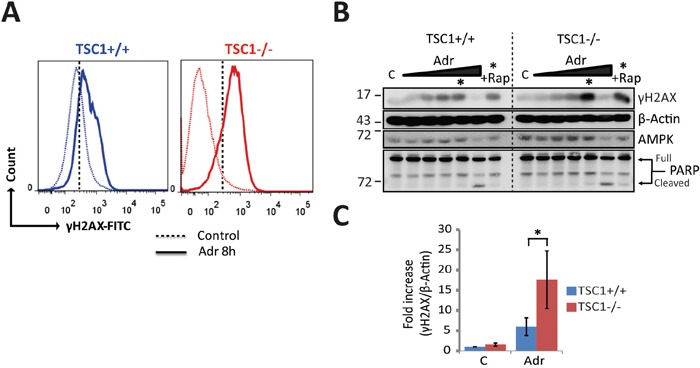
TSC1_-/-_ cells gather primary genetic insults under mild genotoxic stress **A.** Representative histograms of Ser139-phosphorylated H2AX /DNA content (propidium iodide) flow cytometry for DNA damage estimation in TSC1^+/+^ and TSC1^-/-^ MEFs, untreated or acutely treated with 0.5 μg/ml Adr for 8h. Dotted vertical line corresponds to the arbitrary gating threshold also used in Supplementary Figure S6 for illustrative reasons. Note that the data shown here and in Supplementary Figure S6 represent the same experiment. **B.** Dose-response western blot analysis for γH2AX as an indicator of DNA strand breaks of TSC1^+/+^ and TSC1-/- MEFs after Adr treatment for 20 h. Treatment range included 0.01, 0.05, 0.1, 0.5 and 10 μg/ml respectively. Asterisk denotes the 0.5 μg/ml Adr point, and +Rap indicates rapamycin co-treatment. Notice the clearly higher phosphorylation levels of H2AX indicating higher DNA damage accumulation. Weak protein signals at 10 μg/ml reflect poor protein recovery due to collossal cell loss, evident from cleaved PARP. **C.** Densitometry of γH2AX western blots from 3 independent experiments indicating higher phosphorylation levels in TSC1^-/-^ MEFs after Adr treatment. Values are mean ± SD. Statistical significance was calculated using the non-parametric Mann-Whitney U test.

Given this extreme hypersensitivity to DNA damage, we wondered if such hypersensitivity to genotoxic stress is due to an exacerbated DNA damage response or to the accrual of elevated levels of primary genetic lesions. Estimating S139-phosphorylated H2AX (γH2AX) as a DNA damage marker after acute Adr treatment (8h), we found TSC1^-/-^ MEFs gathered significantly higher levels of DNA strand breaks as assessed by flow cytometry (Figure 2A and Supplementary Figure S6), or western blotting (Figure 2B, quantification in Figure 2C). This differential sensitivity to genotoxic stress of wt and TSC1^-/-^ cells was best seen in a window of Adriamycin concentrations around 0.5 μg/ml. Collectively these data establish TSC loss as a factor predisposing tumour cells to DNA damage and cell death in a background of mild genotoxic stress.

### TSC1^-/-^ MEFs feature cell cycle alterations

**Figure 3:**
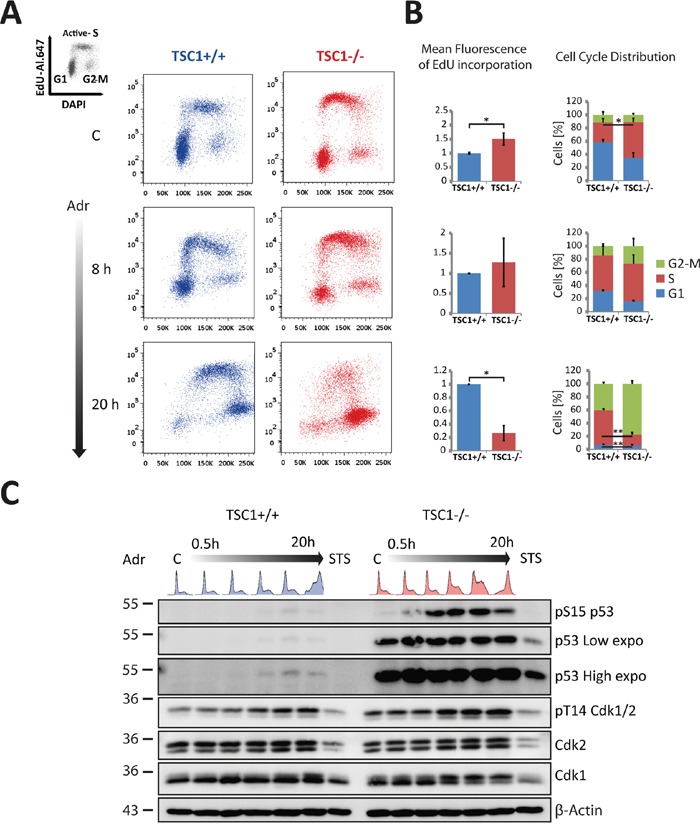
TSC1^-/-^ MEFs feature altered cell cycle distribution, aberrant S-phase progression and G2-M accumulation under mild genotoxic stress **A.** Representative EdU incorporation cell cycle profiles of TSC1^+/+^ and TSC1^-/-^ MEFs untreated or Adr (0.5 μg/ml) treated for the indicated time-periods. The complete time series is shown in Supplementary Figure S7A. **B.** Quantification of mean EdU incorporation intensities (left) and mean cell cycle distribution (right) corresponding to A. Notice the recovery of the S-phase arc in the wt MEFs as opposed to the chaotic S-phase arc in TSC1^-/-^ MEFs at 20 h accompanied by the massive G2-M arrest. Over 75 % of TSC1^-/-^ pass through S-phase and accumulate in G2/M. Values are mean + SD. Statistical significance was calculated using two-tailed t-test. * p<0.05, ** p<0.01. A complete series of EdU incorporation profiles for all treatment times is presented in Supplementary Figure S7A. **C.** Western blot analysis of samples treated for up to 20 h with Adr (0.5 μg/ml). A long exposure of the p53 western is shown to illustrate that p53 does accumulate in wild-type MEF cells, too. Cell cycle patterns are highlighted on top of the lanes to assist interpretation of the blots. STS: Staurosporine (1 μM) serves as a positive control for apoptosis induction.

Exorbitantly high mTORC1 activity is a characteristic feature of TSC null cells that probably mediates most of the phenotypes observed in TSC mutant cells. Beyond being best known as a metabolic master controller, mTORC1 has been uncovered as a genuine cell cycle regulator [27]. Constitutive activity of the pro-anabolic mTORC1 pathway is known to drive premature S-phase entry [22]. DNA replication is the most vulnerable process predisposing cells to DNA damage, genomic instability and cancer [28]. To verify the extent of derangement in cell cycle distribution and how this might affect sensitivity of TSC1^-/-^ MEFs to mild genotoxic stress, we followed TSC1^+/+^ and TSC1^-/-^ MEFs under basal conditions and after low dose Adr treatment using pulse EdU incorporation for up to 24h combined with flow cytometry (Supplementary Figure S7A, for gating strategy see Supplementary Figure S8). An increased S-phase proportion characteristic of tumour cells was accompanied by a striking, significant increment in the peak EdU incorporation marking higher peak DNA synthesis rates in TSC-/- cells (illustrated for representative time points in Figure 3A, quantification in Figure 3B). The larger S-phase populace suggested on the other hand an apparently slower progressing S-phase in TSC1^-/-^ MEFs under basal cycling conditions and, at first glance, was hard to reconcile with the particularly high DNA synthesis rates. Moreover, under Adr treatment, we observed incessant nucleotide incorporation in TSC^-/-^ cells, as opposed to a tangible decline in the EdU “arcs” of TSC1^+/+^ MEFs (see Supplementary Figure S7B), ultimately culminating in a catastrophic S-phase and a massive G2-M arrest (Figure 3A, 8h and 20h, compare also time course in Supplementary Figure S7A). Cell cycle arrest was manifested by p53 stabilisation and activation (Ser15-phosphorylated p53) and elevated inhibitory Cdk phosphorylation at threonine 14 across the time course (Figure 3C). We also note that the S-phase Cdk2 levels are marginally down-regulated by 16 % in TSC1^-/-^ MEFs (Supplementary Figure S9). Thus, TSC loss elevates global nucleotide incorporation rates, accelerates G1 to S transition, and alters cell cycle distribution and kinetics under external genotoxic stress. Together, we conclude that TSC loss directly or indirectly renders cells resilient to cell cycle arrest in G1 or S phase, leading to damage-prone progression through S-phase and a massive G2/M arrest upon genotoxic challenge.

Since TSC1^-/-^ cells have high numbers of cells in S-phase beforehand and given their premature and hasty entry into S-phase in a background of wide-spread DNA damage we wondered if TSC1^-/-^ cells were particularly prone to dying during replication in S-phase. To address this possibility, we monitored cell death via PI and Annexin-V staining along all time points of the Adr time course shown in Figure 3A and Supplementary Figure S7A. This experiment, shown in Supplementary Figure S10, showed no evidence of increased cell death during S-phase but rather confirmed that TSC1^-/-^ cells die following entrance into G2/M. Taking all findings together we conclude that mild DNA damage infringed by Adr drives TSC1^-/-^ cells precipitously into G2/M at which point they succumb to an atypical, largely p53-independent type of death.

### TSC loss perturbs DNA replication

**Figure 4:**
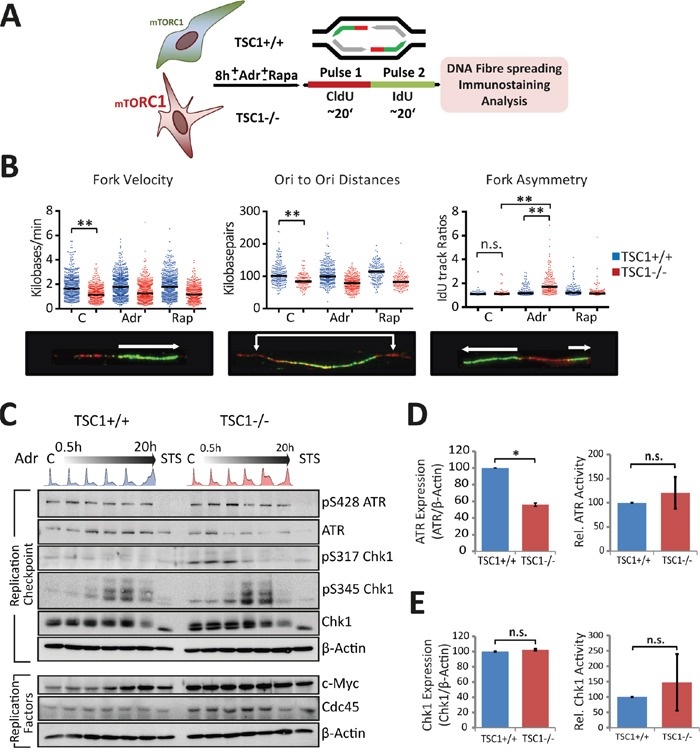
TSC loss perturbs S-phase progression **A.** Schematic of the pulse labelling protocol for fibre assay. See experimental section for details. **B.** Panel shows fork velocity (kb/min), Ori-Ori distances (Kb), and fork asymmetry (IdU track ratios). Dots indicate individual measurements from 3 independent experiments. Statistical significance was calculated using one-way ANOVA (non-parametric Kruskal-Wallis test). *p<0.05, **p<0.01, ***p<0.001, n.s.- not significant. **C.** Western blot analysis of S-phase checkpoint response and replication proteins in TSC1^+/+^ and TSC1^-/-^ MEFs treated with 0.5 μg/ml Adr over lengths of time up to 20 h. STS: Staurosporine. **D, E.** Densitometry for relative expression and activity levels of the replication checkpoint kinases ATR and Chk1 respectively, in untreated TSC1^+/+^ and TSC1^-/-^ MEFs from six independent experiments. Data represent mean ± SD. Statistical significance was estimated using the non-parametric Mann-Whittney U –test. *p<0.05.

Elevated peak nucleotide incorporation rates and yet an over-representation of S-phase cells in cycling TSC1^-/-^ cells intrigued us, since rapid DNA synthesis should intuitively lead to faster progression through S-phase, as typically seen in cancers [29]. To clarify the impact of TSC loss on S-phase control, we wished to inspect replication properties in TSC1^-/-^ MEFs in more detail using the dual pulse-labelling DNA fibre assay [30] (Figure 4A). To our surprise, we observed that in TSC1^-/-^ cells, replication forks progressed significantly slower than their wild-type counterparts (Figure 4B). Neither low levels of Adr nor mTOR inhibition affected fork progression rates of the wt or mutant forks, respectively. The higher overall EdU incorporation rates (Figure 3A) and reduced fork velocity in TSC1^-/-^ MEFs (Figure 4B) could only be reconciled by invoking a higher use of origins (ori) in the TSC mutant cells. Indeed, we observed an overuse of licenced origins as indicated by the reduction in ori-to-ori distances in TSC1^-/-^ MEFs (Figure 4B). Although these features (the reduced fork progression rates to meet the excessive origin firing) are typical for DNA replication stress [31, 32] (reviewed in [29]), twin forks progressing from the same origin were virtually free of any spontaneous asymmetry (Figure 4B). Thus, TSC1^-/-^ cells seem to lack fork stalling under undisturbed growth condition, which commonly accompanies replicative stress driven e.g. by oncogenes such as Ras, c-Myc or cyclin E [33, 34]. However, under acute (8h) mild genotoxic stress, TSC1^-/-^ cells gathered gross asymmetry, reiterating that TSC loss renders cells sensitive to primary DNA damage (Figure 4B). In contrast, ori-to-ori distances and fork progression rates were not significantly affected by the low concentrations of Adr or Rap. Based on the potential replication stress phenotype, we elaborated on the levels of critical DNA replication regulators. In fact, we found an unusual down-regulation of ATR kinase (Figure 4C, 4D) in TSC1^-/-^ cells, despite at least equivalent levels of activated ATR auto-phosphorylated at S428. Chk1 expression levels were relatively unaltered (Figure 4C, 4E). Also, phospho-Chk1 levels were not significantly altered in TSC1^-/-^ MEFs compared to wt controls (as monitored for the two major Chk1 phosphorylation sites S317 and S345; Figure 4C, 4E). Interestingly, we further observed higher, albeit not statistically significant, c-Myc and Cdc45 expression levels in TSC1^-/-^ MEFs (Figure 4C), hinting to a possible role for exacerbated c-Myc activity in conjunction with Cdc45 in driving S-phase entry and promoting DNA replication initiation in those cells. Thus, while TSC^-/-^ cells do exhibit certain features of traditional replicative stress, they do lack others such as replication fork asymmetry rendering the observed phenotype an unconventional type of replication anomaly.

### Energetic enrichment alleviates DNA damage in TSC1_-/-_ MEFs

**Figure 5:**
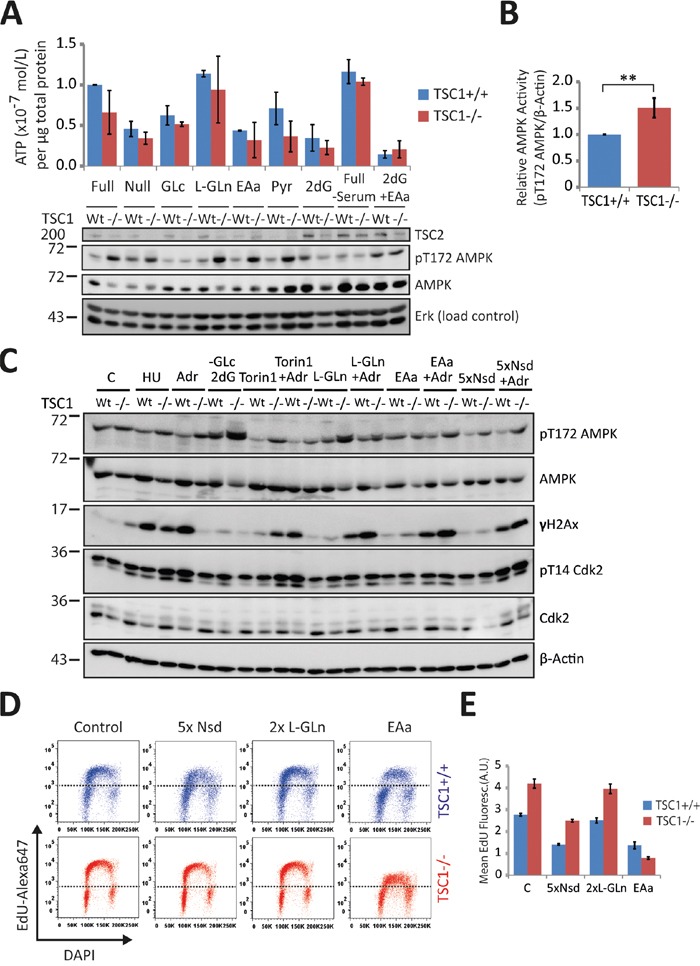
Energetic enrichment in TSC1^-/-^ MEFs alleviates DNA damage accumulation **A.** Above – Luminometric ATP measurements of TSC1^+/+^ and TSC1^-/-^ MEFs under diverse growth conditions as indicated for 20 h. Below – Western analysis of duplicate samples. Note that AMPK activity, scored here as phosphorylation at Thr172, reflects the drop in ATP levels, and is consistently high in TSC1_-/-_ MEFs. **B.** Densitometry of AMPK activity in untreated TSC1^+/+^ and TSC1^-/-^ MEFs maintained in complete DMEM supplemented with 10 % serum. Notice the higher phosphoT172-AMPK levels (activity) due to the increased anabolic demand imposed by constitutive mTORC1 signalling in TSC1^-/-^ MEFs. Bars are mean ± SD. Statistical significance was calculated using the non-parametric Mann Whitney U test. **p<0.01 **C.** Western blot of TSC1^+/+^ and TSC1^-/-^ MEFs cultured for 8h in the presence of the indicated media/supplements. Note that energy deprivation alone does not manifest as spontaneous DNA damage in TSC1^-/-^ MEFs. GLc: Glucose, 2dG: 2-deoxy-Glucose, L-Gln: L-Glutamine, EAa: Amino acids, Nsd – Nucleosides. **D.** Pulse EdU-incorporation cell cycle profiles of TSC1^+/+^ and TSC1^-/-^ MEFs subjected to nucleoside supplementation (5xNsd), high-energy substrate-feeding (2xL-Gln) or amino acid feeding (EAa). Dotted black line is arbitrarily placed to aid visualisation of the changes in EdU-incorporation arc heights **E.** Mean fluorescence of EdU incorporation. Data represent duplicate measurements from one experiment.

Increased energy demand has previously been shown to negatively impact TSC^-/-^ cell survival under metabolic stress [14, 35, 36]. AMP-activated protein kinase (AMPK) is the master energy sensor coordinating cell growth with ATP sufficiency [37, 38]. Consistent with previous work, we observed an overall higher ATP consumption in TSC1^-/-^ cells under a diverse range of growth conditions and energy sources (Figure 5A) suggesting that a general energetic insufficiency may exacerbate several readouts of genotoxic stress-induced death in our studies. Such trend of lower ATP levels is also concordant with AMPK activation states (Thr172-phosphorylated AMPK) in TSC1^-/-^ cells (Figure 5A, 5B). We observe that in the absence of all other nutrient sources, L-Glutamine serves as a sustained energy source, while essential amino acid feeding leads to a drastic decline in ATP levels. L-Glutamine, by way of ‘anaplerosis’ [39] has been shown to protect tumour cells from starvation and metabolic stress-induced death; in fact Glutamine alone is known to sustain cell viability for extended periods of time *in vitro* [14, 40, 41]. We then sought to evaluate how energy limitation or availability influences the sensitivity of TSC1^-/-^ cells to mild genotoxic stress. Hence we performed western analysis with a range of manipulations (Figure 5C) all in the absence or presence of DNA damage imparted by 8 h incubation with Adr. We found that while energy deprivation alone (-Glc, 2DG; glucose free medium plus the glycolysis poison 2-deoxyglucose) did not manifest as DNA damage, increasing the energy expenditure (EAa; essential amino acid feeding, augmented protein synthesis) elevated H2AX S139-phosphorylation, more so in presence of external genotoxic stress. These data indicate that energy shortage synergises with genotoxic agents in causing DNA damage. On the other hand, limiting energy consuming processes (Torin1, mTORC1 inhibition) or supplementing TSC1^-/-^ cells with high energy substrates (L-Gln, anaplerotic and Nsd, nucleoside feeding, thereby also relieving possible nucleotide shortage) only marginally alleviated DNA damage in presence of genotoxic stress (Figure 5C). Looking at EdU-incorporation S-phase arcs following high energy substrate-feeding, we found that nucleoside levels do not pose a restriction to DNA synthesis of TSC1-/- cells (Figure 5D, quantification in Figure 5E). The contrasting, simultaneous decline in mean EdU fluorescence intensity in both TSC1^+/+^ and TSC1^-/-^ MEFs, we attribute to a competition-based dilution of EdU labelling following nucleoside-feeding, despite experimental care. Strikingly, amino acid supplementation led to a drastic collapse of DNA synthesis, as illustrated by the drop in S-phase EdU-arc fluorescence (Figure 5D, 5E), corroborating once more that energy expenditure compromises faithful and undisturbed DNA replication. In conclusion, we postulate that the diminished fork velocity in TSC1^-/-^ MEFs reflects an unmet energy demand for DNA synthesis as a consequence of subversion to other cytoplasmic processes impelled by a pro-anabolic status, probably as a result of high mTORC1 activity.

### G2-M checkpoint infidelity and mitotic catastrophe in TSC1^-/-^ MEFs

**Figure 6:**
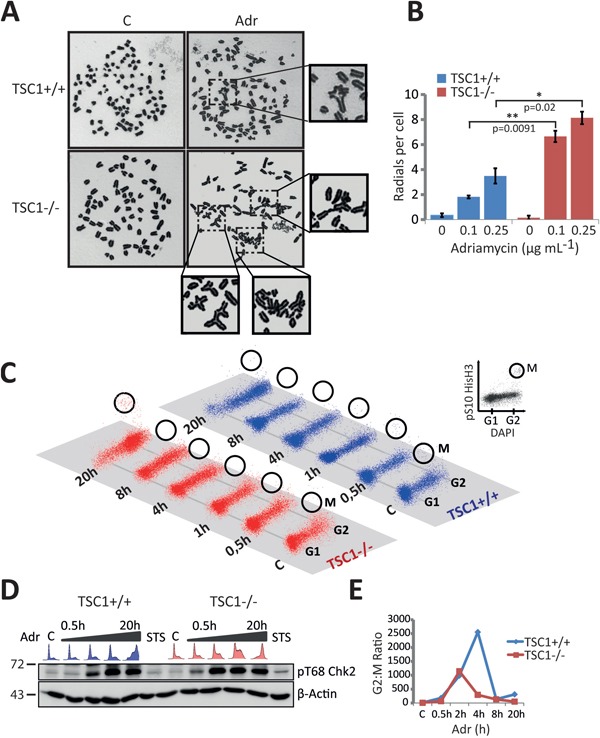
Leaky G2-M checkpoint and catastrophic cell death in TSC1^-/-^ MEFs **A-B.** Representative metaphase chromosome spreads and quantification indicating frequency of aberrations following low-dose Adriamycin treatment per 100 spreads from two independent experiments. **C.** Mitotic entry monitored at various time-points after 0.5 μg/ml Adriamycin treatment in TSC1^+/+^ and TSC1^-/-^ MEFs, by DAPI/pSer10–HisH3 flow cytometry to distinguish between G2 and M phase cells. **D.** Western blot showing Chk2 activation, indicating a proficient ATM-Chk2-mediated G2-M checkpoint after Adriamycin damage. **E.** Percentage ratios of G2 to M phase cells as a measure of the fidelity of G2/M checkpoint, plotted as the geometric mean of 2 experiments; the lower ratios in TSC1^-/-^ MEFs suggest a checkpoint maintenance defect, eventually permitting damage-prone mitotic entry. Representative flow cytometry gating strategy is presented in Supplementary Figure S8. Raw data are provided in Supplementary Table S1.

The G2/M checkpoint prevents mitotic entry of cells with under-replicated or damaged DNA. While the G2/M checkpoint is predominantly governed by the ATM-Chk2 pathway [42-44], the ATR kinase is known to coordinate chromosome condensation with nuclear envelope breakdown [45]. In the light of ATR down-regulation (Figure 4C, 4D), since we observed both an exalted cell death (Figure 1B) and a chaotic S-phase population accompanied by massive G2-M arrest (Figure 3A, 3B) after 20h exposure to Adr in TSC1^-/-^ MEFs, we questioned the possibility of a mitotic catastrophe and pursued investigating the fidelity of the G2-M checkpoint control. Firstly, metaphase chromosome analysis yielded a significantly higher number of radial chromosomes following low-dose Adr treatment in TSC1^-/-^ MEFs (Figure 6A, 6B). Radial chromosomes are an abnormal chromosome structure that results from asymmetrical exchanges of non-homologous chromatids during S phase [46]. These structures are commonly observed in chromosome spreads prepared from cells with underlying predisposition to chromosome instability, such as cells from patients with Fanconi anaemia, Bloom syndrome or ataxia telangiectasia [47, 48]. Secondly, in similar time-course experiments under Adr treatment using Ser10-phosphorylated histone H3 (Figure 6C) in addition to EdU-Alexa647 and DAPI, we monitored the strength of the G2-M checkpoint. While western blot analysis of Chk2 phosphorylation pattern indicated functional G2-M checkpoint responses (Figure 6D), the lower ratio of G2 to M phase cells hinted at a leaky checkpoint in effect leading to promiscuous, damage-prone mitotic entry of TSC1^-/-^ MEFs (Figure 6E, gating strategy shown in Supplementary Figure S8 and raw data in Supplementary Table S1). Thus an increased propensity to gather genetic insults under mild genotoxic stress, coupled with an infidel G2-M checkpoint drives TSC1^-/-^ cells into mitotic catastrophe.

## DISCUSSION

TSC mutant cells exhibit extreme sensitivity to mild stress of various kinds [12, 49-53], a feature that has been attributed to metabolic aberrancies resulting from the exuberantly high mTORC1 activity in TSC^-/-^ cells [14]. More recently, a function of mTORC1 as a cell cycle regulator became evident [22, 27, 54]. Here, we probed the nature and outcome of TSC loss on cell cycle progression and regulation and how such alterations predispose TSC1^-/-^ cells to mild genotoxic stress-induced death. We find, consistent with previous work, that TSC loss strongly sensitises tumour cells to otherwise harmless doses of genotoxic stress. We observed that, irrespective of the particular treatment regimens which included two different genotoxins and/or acute mTORC1 inhibition, TSC1^-/-^ cells consistently fare worse than their wild-type counterparts. Of interest, TSC tumour cells are predisposed to primary genetic insults despite enhanced p53 expression and stabilisation and proficient cell cycle checkpoint signalling. In line with data from Guan and co-workers [12] we detect elevated levels of p53 in TSC1^-/-^ cells. However, while pharmacological mTORC1 inhibition reduces p53 protein and activity levels it does not alleviate the stress-induced death toll of TSC1^-/-^ cells. The inability of Rapa or of the ATP-competitive mTOR inhibitor Torin1 to protect TSC null cells from stress-induced death is puzzling but not unprecedented, as rapa reportedly also fails to protect TSC null cells against ER stress [50, 55]. This contrasts the ability of Rapa to rescue TSC1-/- cell death induced by glucose withdrawal or DNA damage caused by the alkylating agent MMS [12], evidencing that different mechanisms are at play downstream of distinct genotoxic and metabolic insults. At first sight, the lack of efficacy of mTORC1 inhibition in reverting the TSC null phenotype described here and elsewhere suggests that downstream targets of TSC and/or its substrate G-protein Rheb other than mTORC1 are responsible for the lethal replicative stress phenotype. While a handful of such alternative TSC/Rheb downstream targets have been described [56, 57], an overwhelming body of literature argues for mTORC1 as the major effector of TSC signalling. It appears reasonable to hypothesize that acute mTORC1 inhibition may not suffice to revert a lethal stress response that may require processes lagging well beyond the drop in mTORC1 kinase activity, like e.g. the restoration of the cellular energy balance and/or of physiologically balanced levels of replication factors. In the sum of these considerations, we propose that mTORC1 is the likely mediator of the TSC phenotype described in the current study, although the involvement of other downstream mediators can certainly not be excluded.

With respect to the role of p53, we conclude that while being probably involved in the commitment to stress-induced death in some instances [12], p53 is neither the sole nor dominant cue dictating the downfall of TSC1^-/-^ cells in response to genotoxic stress, at least under the conditions studied here. This notion is reinforced by the observation that a large fraction of death events do not resemble canonical suicidal apoptotic programmes (Supplementary Figure S5), as commonly elicited by p53. Of note, death of TSC1^-/-^ cells in response to mild stress is not mediated by processes converging on DNA damage since death rates do not strictly correlate with DNA damage as scored by H2AX phosphorylation. For example, glucose withdrawal and/or a block in glycolysis elicit a strong drop in ATP levels (Figure 5) and extensive cell death [12] yet do not induce DNA damage (Figure 5). Also, pharmacological mTORC1 inhibition restores ATP levels [14] (Figure 5C) and alleviates Adr-induced DNA damage (Figure 5C), yet does not protect TSC1^-/-^ cells from genotoxic stress-induced death (Figure 1B). Thus, TSC1^-/-^ cell death from energy insufficiency does not reflect an accumulation of DNA lesions due to inefficient maintenance of DNA integrity and is probably mechanistically distinct to the genotoxic stress-induced death investigated here. We conclude that multiple cues (energy insufficiency, high p53 activity, exacerbated DNA damage and possibly the up-regulation of replication factors like Myc) are likely co-contributing factors to the hypersensitivity to genotoxic stress in TSC null cells but none of them stands out as a the single primordial trigger.

A primeval step in tumorigenesis is oncogene-induced replication stress [29, 58]. We document that TSC loss appears to drive a replication phenotype, somewhat ineffectual and distinct from canonical replicative stress (Supplementary Table S2). It is characterised by significantly slower progressing forks but only a marginal origin overuse, and accompanied by down-regulation of the replication checkpoint kinase ATR. In the light of the potentially high c-Myc and Cdc45 expression levels in TSC1^-/-^ cells, this phenotype could seemingly be c-Myc-driven, which is a known translational target of mTORC1 [32, 59]. Of interest, recent reports establish c-Myc as a genuine replication initiation factor, and demonstrate its importance in a direct, non-transcriptional mode of controlling DNA replication [60]. Also, Cdc45 overexpression has been shown to phenocopy c-Myc-driven effects on DNA replication [32]. However, the absence of spontaneous fork asymmetry in TSC1^-/-^ cells, in particular, is a striking deviation from classical replicative stress, suggesting that fork progression rates may be hampered by the general ‘energy debility’ in TSC1^-/-^ cells. We hence term this phenotype energy-restricted replication (ERR) and propose that it reflects rather a lack of ATP than diminished dNTP pools (Supplementary Table S2). This would explain the lack of fork asymmetry, and also why nucleoside feeding did not rescue TSC1^-/-^ cells (Figure 4, 5). In other words, forks seem to progress constantly on “low gas”, rather than by “stop and go”. Our results thus causally implicate energetic compromise in TSC1^-/-^ cells to the previously observed lengthy S-phase in TSC patient material [23]. We hypothesize that the over-firing of replication origins resulting in higher EdU incorporation rates in TSC1^-/-^ MEFs is possibly an outcome of enhanced c-Myc translation by mTORC1, thus enhancing the impact of energy depletion on DNA replication. Thus, ERR in combination with energy insufficiency could work together to elicit the observed unconventional replication phenotype. This does not lead to increased Chk1 levels or phosphorylation, and does not trigger genomic instability *per se*, as judged by a lack of H2AX phosphorylation (Figure 1C, 5C) and chromosomal aberrations in the absence of exogenous genotoxic stress (Figure 6A, 6B). DNA helicases in particular are highly ATP-dependent. Our observation of Cdc45 up-regulation in TSC1^-/-^ cells suggests that CMG helicase complex formation is increased, and DNA unwinding may become rate-limiting for the forks. Since anaplerotic ATP-provision via glutamine supplementation fails to revert EdU-incorporation despite alleviating ATP scarcity (Figure 5), energy sufficiency alone is probably not sufficient to revert replicative stress driven by other mTORC1-dependent cues, e.g. high c-Myc activity.

In sum, our data suggest that increased anabolic demand and energetically impoverished milieu in TSC1^-/-^ cells act to their detriment under stress conditions. Inappropriate consumption or diversion of cellular energy for mTORC1-controlled anabolic pathways viz., protein translation could limit energy input for DNA damage response, repair reactions and/or chromatin remodelling, leading to DNA damage persistence and the augmented use of dormant origins during undisturbed growth. This scenario rationalizes how even low levels of DNA damage trigger the catastrophic genome instability in TSC-/- cells reported here.

**Figure 7:**
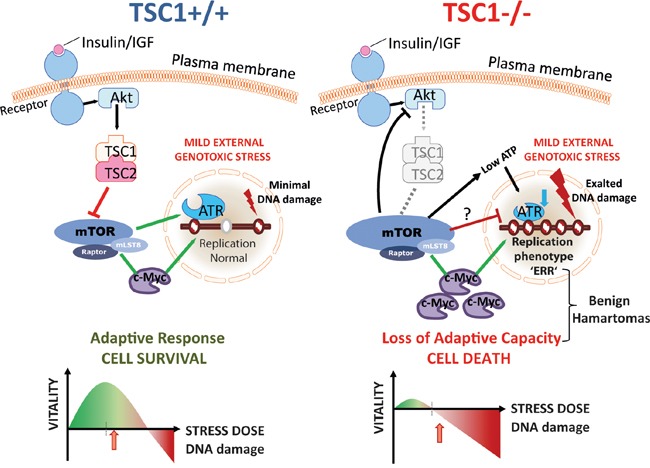
mTORC1, genotoxic stress response and tuberous sclerosis Model summarising various factors converging in the loss of mild stress adaptation in TSC1^-/-^ cells. A constitutively anabolic state with increased energy expenditure, perturbed cell cycle progression including S-phase checkpoint kinase–ATR downregulation, a restrained replication phenotype with modest origin over-use and declined fork progression rates, high p53 levels, altogether set stage for a failure of adaptation of TSC1^-/-^ cells to mild external stress doses, given the inherent stress milieu (also see Supplementary Table S2).

TSC tumours are virtually devoid of karyotypic alterations, with the exception of rare case reports [61, 62]. Based on the findings reported here, we propose that exacerbated damage accrual and heightened (genotoxic) stress sensitivity, slower DNA synthesis and elevated p53 function co-operate in preventing genomic instability and complete genome replication, thus precluding malignant transformation and setting stage for an inherent negative selection within the TSC tumours (Figure 7). This in our view offers first-hand evidence reasoning why TSC is a benign syndrome.

TSC patients present with neurological manifestations including cortical tubers, sub-ependymal nodules and sub-ependymal giant cell astrocytomas (SEGAs), epilepsy and mental retardation [10, 11]. Rapa and certain rapalogs have proven successful only in trials against a subset of mTORC1-hyperactive tumours. Interestingly, this effect has been attributed to the effective inhibition of the S6K but not the 4E-BP1 signalling branch downstream of mTORC1 [63, 64]. Insights into the role of hyperactive mTORC1 in cell cycle progression may open a paradigm for targeted therapies aimed at selective killing of TSC tumours, taking advantage of the RS-like stress hypersensitivity to otherwise harmless chemotherapeutic doses, although manifestations involving neuronal functions, epilepsy in particular, may require continuous management using an independent treatment modality. In view of the fact that mTORC1 hyperactivity sensitises tumour cells to mild genotoxic stress, and ‘incomplete’ mTORC1 inhibition by Rapa may inadvertently rescue this phenotype employing mild genotoxic stress as an approach may aid in selective elimination of TSC-lacking tumour cells, also overcoming Rapa-refractory tumours.

## MATERIALS AND METHODS

### Cell cultures and treatments

Cellular models of Tuberous Sclerosis Complex (TSC), including TSC1^-/-^ (Hamartin null) and TSC2^-/-^ p53^-/-^ (Tuberin, p53 double null) mouse embryonic fibroblasts (MEFs) along with their Wild-Type (wt) and p53^-/-^ counterparts were procured from the Kwiatkowski laboratory, Boston, MA, and maintained in continuous cultures in Dulbecco's Modified Eagle's Media with stable L-glutamine (DMEM; Sigma, #D6429) supplemented with 10 % FCS under standard conditions (95 % humidity, 5 % CO_2_, 37 °C). Cell populations were regularly checked for the occurrence of aneuploidy by FACS analysis. For various treatments involving growth conditions, cells were cultured in DMEM without D-Glucose and L-Glutamine (Gibco, #A14430) or Dulbecco's PBS (Gibco, #14040-117) supplemented with the indicated nutrient(s), dialysed FCS and carbon energy source. Genotoxic treatments with Hydroxyurea (HU, 2 mM) and Adriamycin (Adr, 0.5 μg/ ml) were for indicated periods as in the relevant results section. Hydroxyurea (HU) (H8627), doxorubicin hydrochloride (Adriamycin) (D1515), CIdU (C6891), IdU (I7125), DAPI (D9542) were procured from SIGMA; Rapamycin (553210) was procured from Calbiochem.; Torin1 (4247) was purchased from Tocris; GlutaMax (35050-038) was procured from Gibco® by Life technologies; MEM amino acids (M11-002) and sodium pyruvate (S11-003) were from PAA Laboratories GmbH; 2-Deoxy-D-Glucose (31066) was from Fluka Biochemika; Glucose (8337 39002-019) was acquired from Merck KGaA Gibco BRL.

### Western blotting

Following indicated treatments, cells were lysed and scraped to resuspension on ice in RIPA buffer (50 mM Tris-HCl pH-8.0, 150 mM NaCl, 5 mM MgCl_2_, 1% NP-40, 0.5 % DOC, 0.1 % SDS). Absolute protein concentrations from whole-cell RIPA lysates were quantified using Pierce® BCA Protein Assay Kit following the manufacturer's instructions (Thermo Scientific^TM^; #23225 and #23227). Based on abundance of specific proteins under study and resolution requirement, 25-50 μg of total protein was resolved on standard or 8-10 % gradient polyacrylamide gels (Lonza, ProSieve, #50618) according to the manufacturer's instructions. Proteins were transferred on to PVDF membranes (Millipore, Immobilon-P, #IPVH00010 and ISEQ00010) and blots were probed as indicated in the figure panels with the following primary antibodies: β–Actin mAb (A5441) from SIGMA; anti-phospho-Histone H2AX (Ser 139) clone JBW301 FITC conjugate (FCMAB 16-202A), anti-phospho-Histone H2AX (Ser 139) clone JBW301 (05-636), anti-phospho-Histone H3 (Ser10), clone 3H10 Alexa Fluor® 488 conjugated (FCMAB104A4) all from Merck Millipore Corporation; p53 (1C12) mouse mAb (2524S), PARP rabbit Ab (9542S), P-p53 (S15) rabbit Ab (9284S), P-AMPK alpha (T172) (40H9) rabbit mAb (2535S) P-p70 S6 Kinase (T 389) rabbit Ab (9205S), p70 S6 Kinase rabbit Ab (9202S), Tuberin/TSC2 (Ser939) (3612S), P-ATR (S248) rabbit Ab (2853S), Chk1 (2G1D5) mouse mAb (2360S), AMPK (2532), P-Chk1 (S345) (133D3), P-Chk2 (T68) rabbit Ab (#2661S) all from Cell Signaling Technology, Inc.; MDM2 (HDM2-323) (sc-56154), Cdk2 (M2) rabbit polyclonal IgG (sc-163), ATR (N-19), Goat polyclonal IgG (sc-1887), rabbit polyclonal c-Myc antibody (N-262) (sc-764) from Santa Cruz Biotechnology, Inc.; rabbit mAb to Cdk2 (phospho T14) [EP 2234Y] (ab68265) was procured from abcam®; rabbit anti-Phospho Chk1 (S317) (A300-163A) was purchased from BETHYL. All antibodies were used at 1:1000 dilution.

### siRNA–mediated acute knockdowns

TSC2 siRNA (Dharmacon, Smart Pool, # L-047050-00-0005) and p53 siRNA (Dharmacon, Smart Pool, #L-040642-00-0005) targeting expression in MEF cells were transfected into WT MEFs and TSC1^-/-^ MEFs using Lipofectamine® RNAiMAX reagent (Invitrogen, Life Technologies #13778) as per the vendors' recommendations. 1.5 μg of the siRNAs were used per well of a 6-well plate with 3.5 – 5 x 10^4^ cells seeded the previous day, incubated for 48-72 h, subjected to treatments and harvested accordingly, for western blotting or flow cytometry.

### ATP measurement

Extracts for ATP measurement were prepared as follows. Briefly, at the end of treatments, MEF cells in 6-well plates were lysed in 0.5 ml of 96 % ethanol, allowed to evaporate (air-dry) or blown-dry, solubilized in 0.5 ml Tris-EDTA buffer (100 mM Tris-HCl, 2 mM EDTA) by freeze-thawing the plate in liquid nitrogen, and the suspension collected with the aid of a cell-scraper into 1.5 ml Eppendorf tubes. Samples were centrifuged at 14,000 rpm for 10’ and the supernatant transferred into fresh Eppendorf tubes. Samples were diluted serially in two steps of 1:25 in a final volume of 800μL prior to determining ATP levels by luciferin-luciferase luminometry employing the kit (Biothema ATP SL, Cat# 144-041) as per the manufacturer's instructions. Duplicate treatments were included for total protein estimation by microBCA assay so as to express ATP levels per μg of total cellular protein and for western blotting.

### Terminal cell death assay by PI exclusion

MEF cells, either untreated or treated as indicated, were harvested by Accutase–treatment and pooled with media supernatant to gather detached, dead cells, centrifuged at 700xg, washed once in cold wash buffer (PBS with 5 % FCS, 4.5g/L D-glucose, MEM vitamins), resuspended in 300 μL of the buffer containing propidium iodide (Calbiochem, EMD chemicals, Inc., #537059) to a final concentration of 1.5μg/ml. Samples were analysed on a FACS canto (BD Biosciences) instrument using the 488-nm laser and the phycoerythrin (PE) channel. Percent PI positive pre-gated singlet cells were accounted as non-viable. FlowJo software was used for analysis and quantification.

### EdU incorporation Click-IT multi-colour flow cytometry

For cell cycle analysis, cells were pulsed with the thymidine analogue 5-ethynyl-2′-deoxyuridine (EdU) for 20’, harvested by Accutase–detachment and stained for DNA synthesis and cell cycle distribution using the Click-iT EdU-AlexaFluor647 Flow Cytometer Assay kit (Molecular Probes, Life Technologies, #C10635), following the manufacturers' protocol. EdU was coupled to AlexaFluor647 azide using standard Copper(I)-catalysis Huisgen 1,3-cycloaddition (click chemistry) and DNA content was determined by 40, 6-diamidino-2-phenylindole dihydrochloride (DAPI, Molecular Probes, Invitrogen, #D1306) staining. In addition, the cells were stained with either anti-phospho-Ser10-histone H3–AlexaFluor488 antibody conjugate (Millipore, # FCMAB104A4) that specifically labels M-phase cells or anti-γH2AX–FITC antibody conjugate (Millipore, #16-202A) as a marker of DNA damage. Samples were subjected to multi-colour flow cytometry on a FACS Canto II (BD Biosciences) cytometer equipped with blue (488-nm), red (633-nm) and violet (405-nm) lasers. The MEF cell population was gated-in with a FSC/SSC dot plot and doublets gated-out based on a DNA dye area/width dot plot. This cell population was further analysed for its cell cycle distribution. G1-, S- and G2/M-phase cell populations were defined in a DNA dye/EdU-Alexa Fluor 647 dot plot and G2/M phase cells were further separated into G2 and M using the DNA dye/AlexaFluor 488 dot plot. FlowJo software was used for analysis and quantification.

### DNA fibre assay

DNA fibres were prepared by on-slide lysis and gravity-spreading as described originally by Jackson and Pombo [65]. Following treatments, exponentially growing MEF cells in either adherent T25 flasks or 60 mm dishes were successively pulse-labelled for 20’ in standard growth medium (DMEM + 10 % FCS) with 25 mM CldU and 250 mM IdU, washed once with ice-cold PBS, and collected by scraping. Roughly 1,000 cells in suspension were lysed in a droplet of 7 μL spreading buffer (200 mM Tris-HCl pH 7.4, 50 mM EDTA, 0.5 % SDS) for 2’ on one end of grease-free microscopic slides and the chromatin spread by slide-tilting and gravity flow of the droplet over several minutes. Once spread and dry, fibres were fixed for 10 min in 3:1 methanol:acetic acid, the slides air-dried, rehydrated, the DNA denatured with 2.5M HCl for 75 min, washed and incubated in blocking buffer (PBS, 1 % BSA, 0.1 % Tween-20) for 1 hr. Fibres were labelled with rat anti-BrdU antibody (1 h, 1:1000, ab6326; Abcam), fixed in 4% PFA for 10 min, and sequentially labelled with anti-rat AlexaFluor 555 antibody (2 h, 1:500; Molecular Probes), mouse BrdU antibody (overnight at 4°C, 1:1,500, 347583; BD), and anti-mouse AlexaFluor 488 antibody (2 h, 1:500; Molecular Probes). Slides were mounted, images acquired under oil-immersion (100X objective) with an Olympus BX61 immunofluorescence microscope and analysed using ImageJ software (http://rsb.info.nih.gov/ij/). CldU and IdU track lengths were measured using ImageJ and appropriate conversions applied for pixels-to-micrometres-to-kilobases as described earlier by Jackson and Pombo. A minimum of 200 replication forks from at least three independent experiments per condition were analysed. Wherever indicated, counts of origins, terminating and elongating structures were determined using the cell counter plug-in for Image J. Fibre tracks were categorised; red-green (ongoing replication), red (stalled or terminated forks), green (2nd pulse origin) and green-red-green (1st pulse origin).

### Metaphase chromosome preparation and aberration assay

For metaphase spreads exponentially growing cells were treated with 0, 0.1 or 0.25 μg/ml Adriamycin for 4h. After medium change cells were treated with colcemid (0.02 μg/ml) overnight, incubated with 0.0075 M KCl, fixed with methanol/acetic acid (3:1), dropped onto microscope slides, stained with 5% Giemsa and mounted with Entellan before imaging with a Zeiss Axioplan 2 microscope. Radial chromosomes of at least 100 metaphases per experiment were counted in two independent experiments and expressed as radial chromosomes per cell. Error bars represent standard error of the mean.

## SUPPLEMENTARY MATERIALS DATA


