# Supplementary material for: TSC loss distorts DNA replication programme and sensitises cells to genotoxic stress
Source: Oncotarget. 2016 Nov 16;7(51):85365–80. doi: 10.18632/oncotarget.13378 (PMC5356742; doi:10.18632/oncotarget.13378)
Supplement: Supplementary file 1 [file oncotarget-07-85365-s001.pdf]

TSC loss distorts DNA replication programme and sensitises cells to genotoxic stress

SUPPLEMENTARY DATA

SUPPLEMENTARY METHOD

Cell cycle stage-specific cell death profiles

MEF cells, either untreated or treated as indicated, were fed with 5µg/mL Hoechst33342 (B2261, SIGMA) live-cell DNA stain for 30mins, harvested by Accutase–treatment and pooled with media supernatant to gather detached, dead cells, centrifuged at 700xg, washed once in cold wash buffer (PBS with 5 % FCS, 4.5g/L D-glucose, MEM vitamins), resuspended in 300 µL of the binding buffer containing Annexin-V-FITC and propidium iodide (BD Pharmingen™) as per manufacturer's recommendations. Samples were analysed on a BD LSRFortessa™ cell analyzer with 355nm, 405nm, 488nm and 640nm laser configuration. Cells pre-gated for singlets were used for Sub-G1 DNA content, percent AnnexinV-FITC positive and percent PI positive cells in G1, S or G2-M stages for analysis of cell death. FlowJo software was used for analysis and quantification.

SUPPLEMENTARY FIGURES

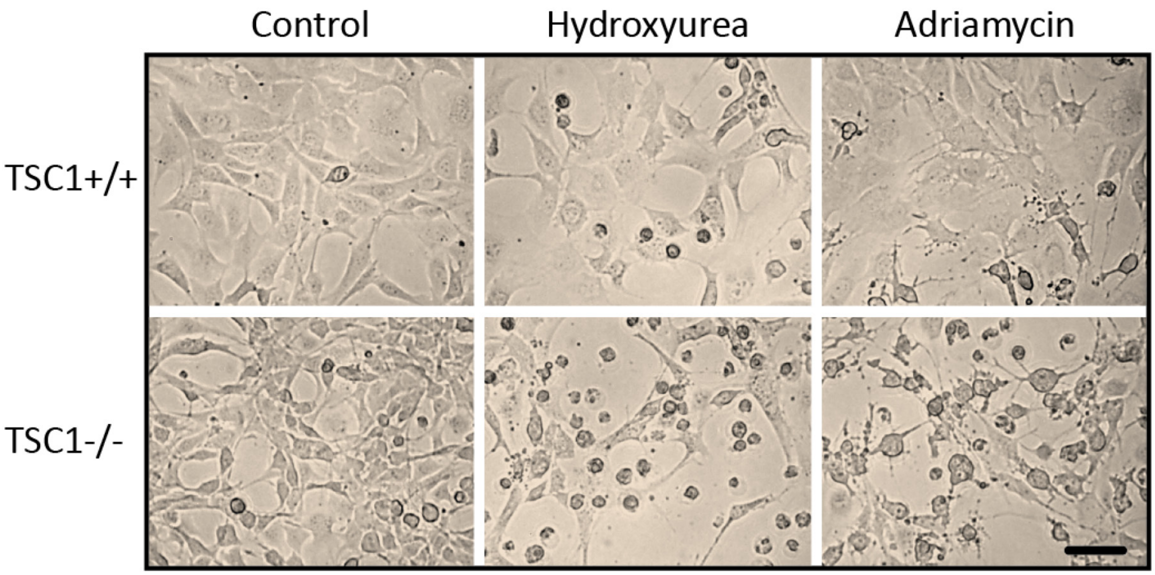

Supplementary Figure S1: TSC1 loss sensitizes cells to genotoxic stress-induced cell death. Light microscopic images of TSC1$^{+/+}$ and TSC1$^{-/-}$ MEFs, untreated or treated with Hydroxyurea (2 mM) and Adriamycin (0.5 µg/ml) respectively, for 20 h. Note the detachment and rounding-off of treated TSC1$^{-/-}$ cells compared to the untreated controls and their wt counterparts. Scale bar = 10 µm.

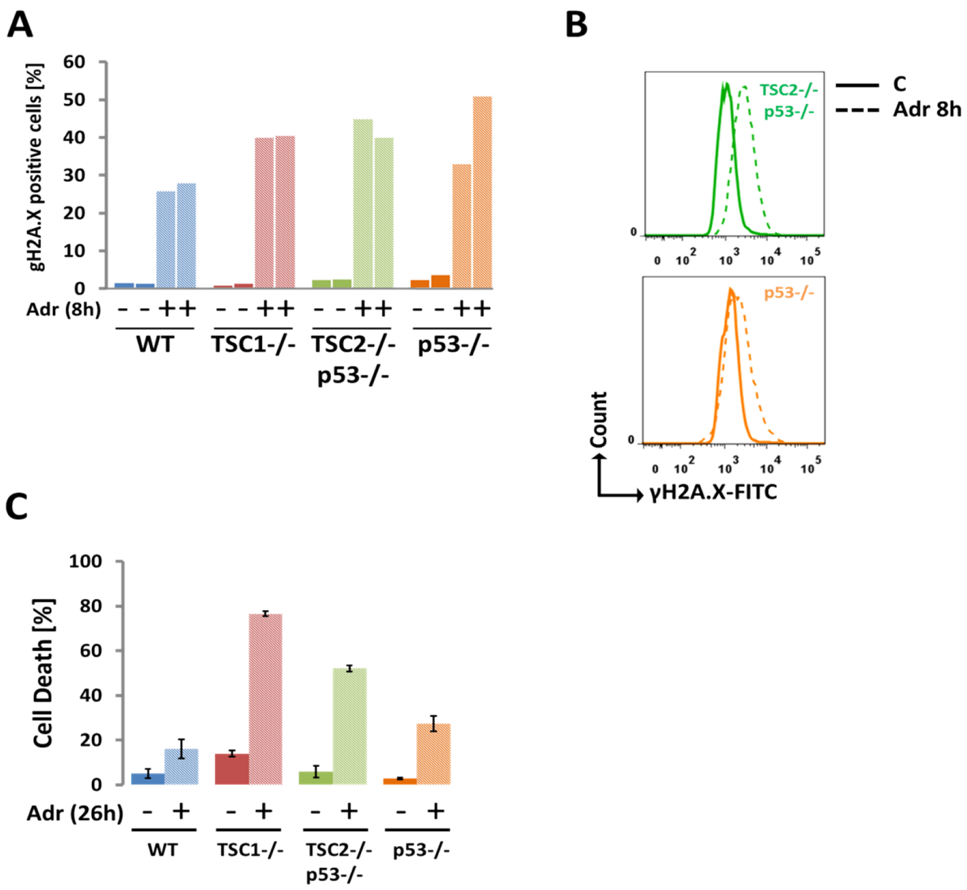

Supplementary Figure S2: TSC2$^{-/-}$ p53$^{-/-}$ MEFs undergo profound cell death following mild genotoxic treatment. A. Ser139-phosphorylated H2AX flow cytometry for DNA damage estimation (n=2), in WT, TSC1$^{-/-}$, TSC2$^{-/-}$p53$^{-/-}$ and p53$^{-/-}$ MEFs untreated or acutely treated with 0.5 µg/ml Adr for 8h. **B.** Representative histograms of TSC2$^{-/-}$p53$^{-/-}$ and p53$^{-/-}$ MEFs from the same experiment. **C.** A typical experiment of propidium iodide (PI) exclusion flow cytometry for cell death quantification. WT, TSC1$^{-/-}$, TSC2$^{-/-}$p53$^{-/-}$ and p53$^{-/-}$ MEFs untreated or treated with Adriamycin (Adr, 0.5µg/mL) for 26h. Data-set are a mean of triplicate samples from one experiment. Error bars represent standard deviation (SD).

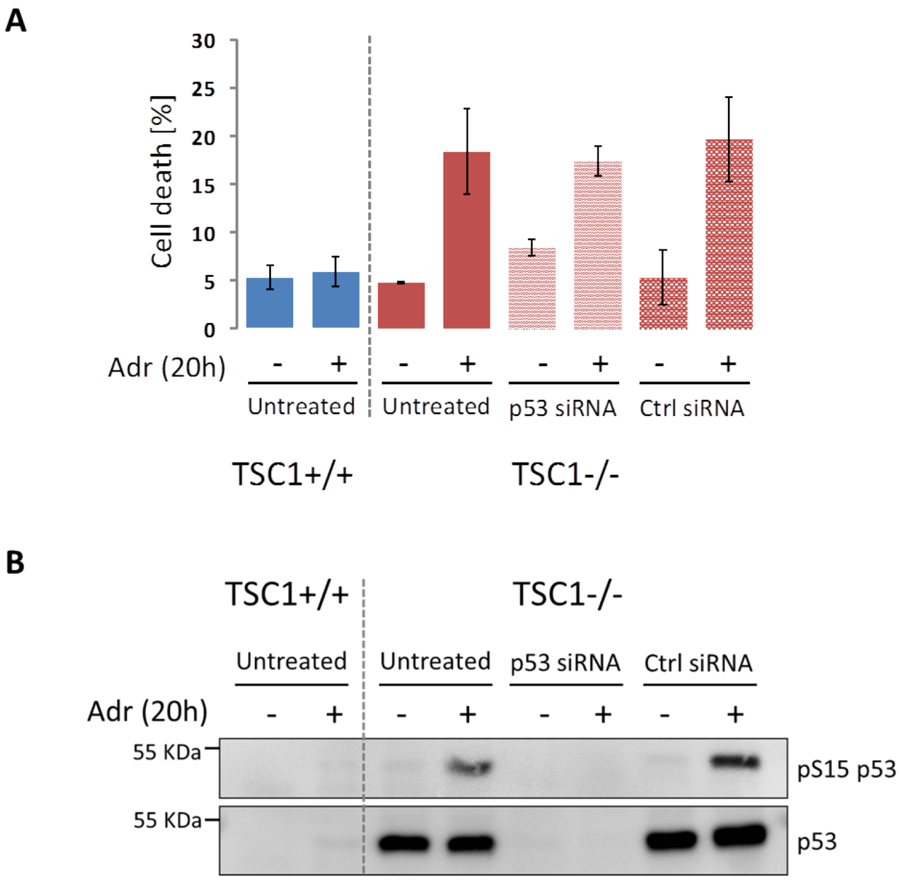

Supplementary Figure S3: TSC1$^{-/-}$ cell death is independent of p53 activity. A. Propidium iodide (PI) exclusion terminal cell death assay for WT and TSC1$^{-/-}$ MEFs untreated or treated with Adriamycin (Adr, 0.5 µg/ml, 20 h) following siRNA-mediated p53 depletion. TSC1$^{+/+}$ MEFs untreated or treated with Adriamycin were used as reference. Data-set are a mean of duplicate samples from two independent experiments. Error bars represent standard deviation (SD). **B.** Representative western blot showing efficiency of p53 knockdown. Non-target control siRNA knockdown neither altered p53 expression nor cell death in TSC1$^{-/-}$ cells.

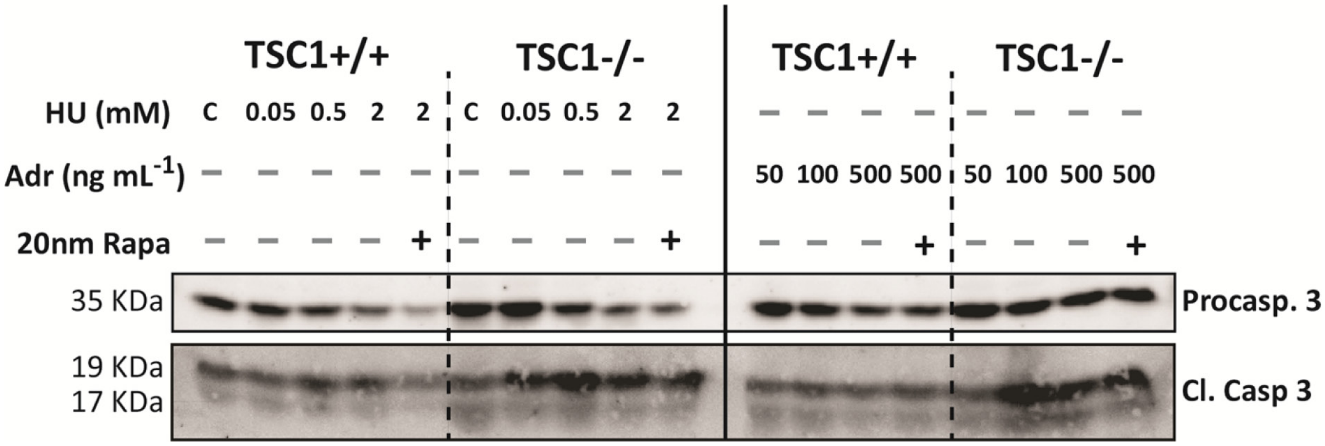

Supplementary Figure S4: Low threshold of DNA damage-induced cell death in TSC1$^{-/-}$ MEFs. Dose-response western blot analysis of TSC1$^{+/+}$ and TSC1$^{-/-}$ MEFs under genotoxic treatments. All treatments were for 20 h. Cleaved caspase-3, an early marker of apoptosis, is far more evident at low doses of genotoxins in TSC1$^{-/-}$ MEFs compared to TSC1$^{+/+}$ MEFs.

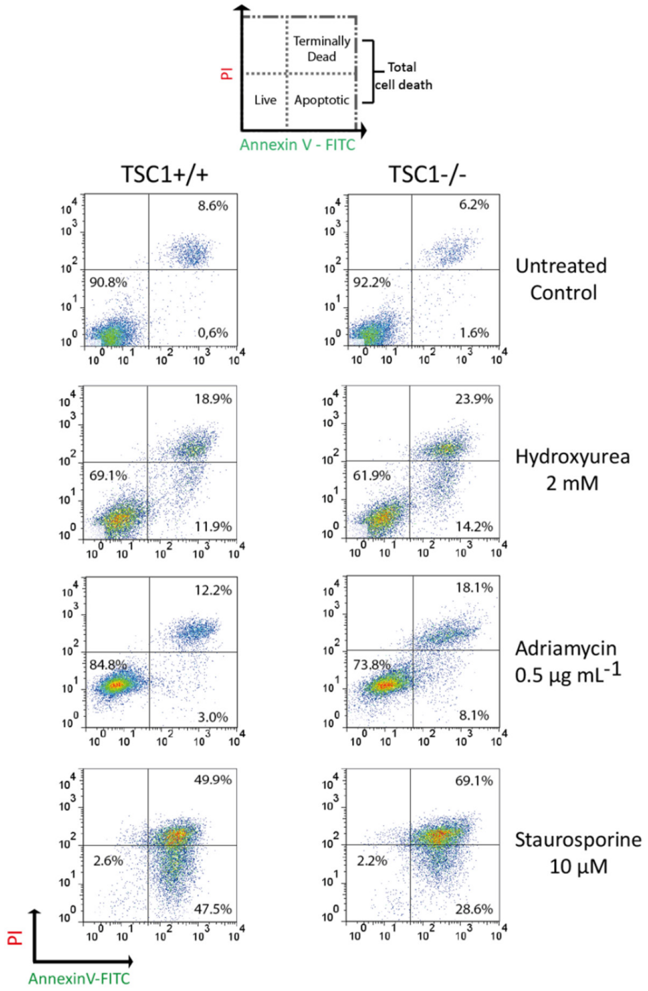

Supplementary Figure S5: Apoptosis is not the sole mode of cell death in TSC1$^{-/-}$ MEFs under mild genotoxic stress. Representative Annexin-V / Propidium Iodide dual labelling live-cell flow cytometry dot-plots of TSC1$^{+/+}$ and TSC1$^{-/-}$ MEFs subjected to HU and Adr treatment. Cell death toll (sum of Annexin-V positive and Annexin-V/PI double positive cells) in TSC1$^{-/-}$ MEFs far exceeds the fraction of bona fide apoptotic (Annexin-V positive) cells, implying that additional cell death mechanisms cannot be ruled out.

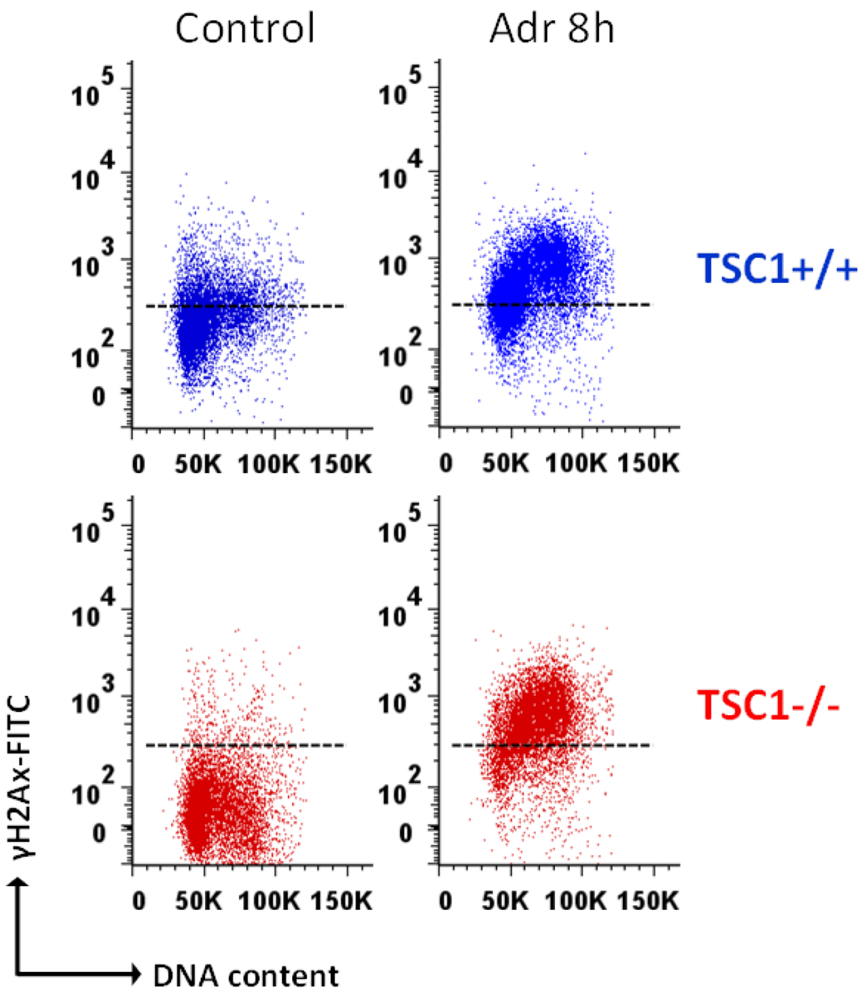

Supplementary Figure S6: TSC1$^{-/-}$ cells gather more primary genetic insults under genotoxic stress. Representative dot plots of Ser139-phosphorylated H2AX /DNA content (propidium iodide) flow cytometry for DNA damage estimation in TSC1$^{+/+}$ and TSC1$^{-/-}$ MEFs, untreated or acutely treated with 0.5 μg/ml Adr for 8h. Percentage of γH2AX-positive events is labelled within the gates. These data and those shown in Figure 2A represent the same experiment.

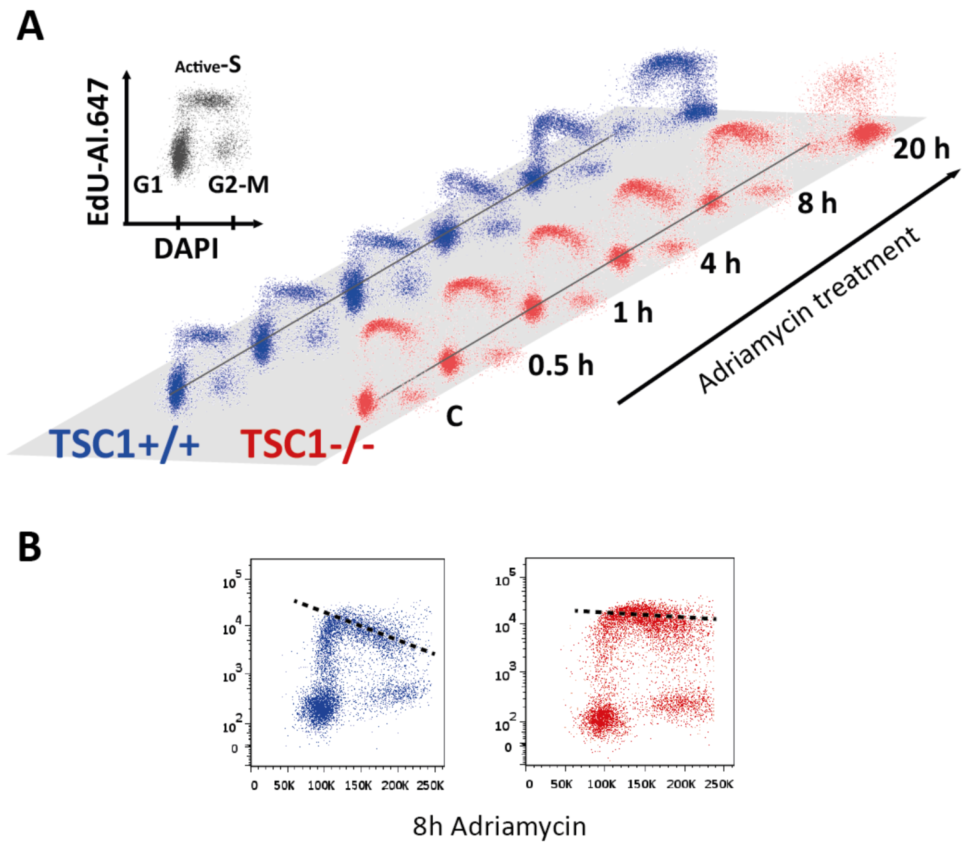

Supplementary Figure S7: Incessant global nucleotide incorporation in TSC1$^{-/-}$ MEFs despite genotoxic stress. A. Representative EdU pulse incorporation flow cytometry cell cycle profiles of TSC1$^{+/+}$ and TSC1$^{-/-}$ MEFs either untreated, or after Adriamycin treatment followed in time for up to 20 h. **B.** Excerpt (8 h points) from the series in A. Observe the decline in nucleotide incorporation in late S-phase at 8 h in WT MEFs compared to the incessant incorporation rates in TSC1$^{-/-}$ MEFs, seemingly unchanged by the presence of the drug. The black dotted line is arbitrary and meant for illustrative purpose only.

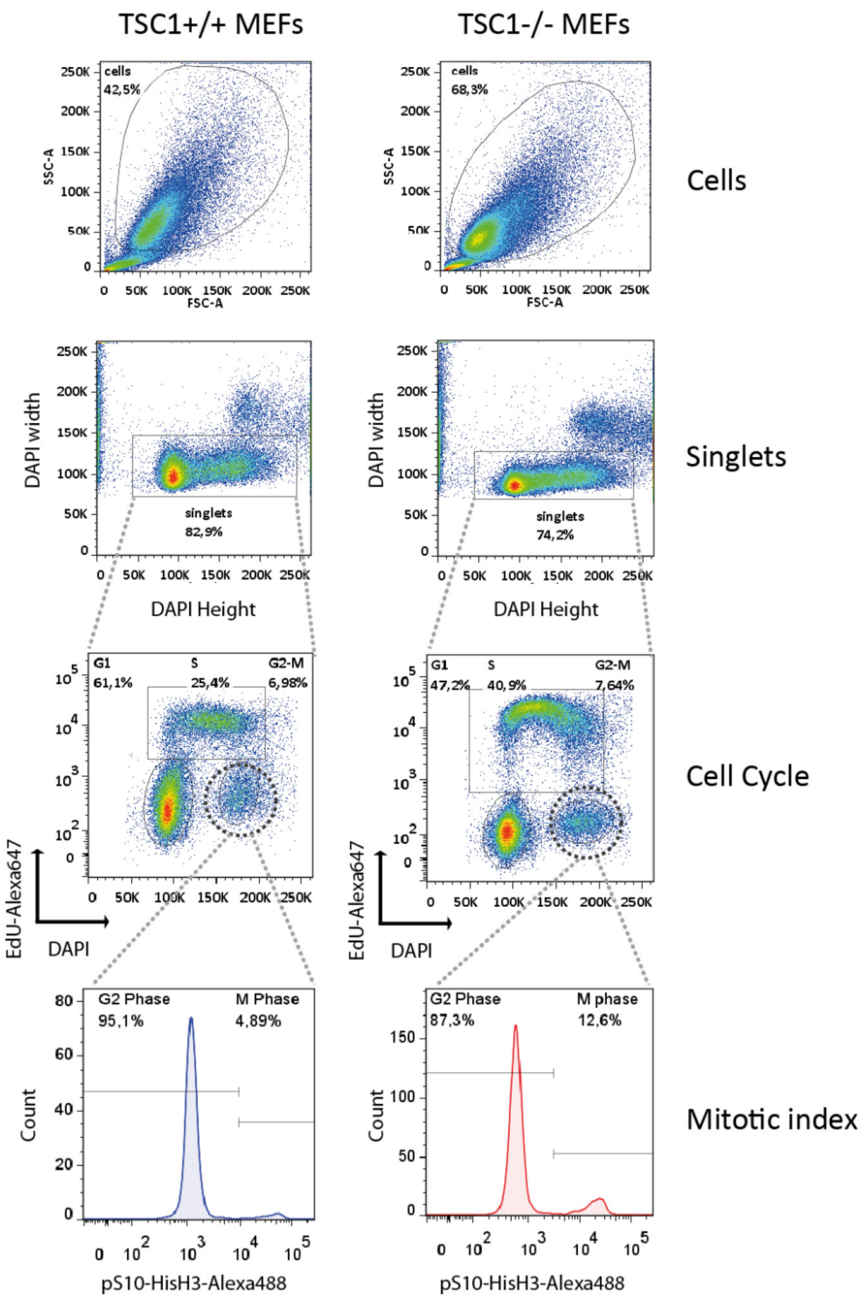

Supplementary Figure S8: Gating strategy for cell cycle profiling and G2-M analyses by Flow cytometry. Gating strategy for EdU pulse incorporation / DAPI / pSer10-HisH3 flow cytometry. Singlets were used for the cell cycle analysis and the G2-M subpopulation to estimate the mitotic index.

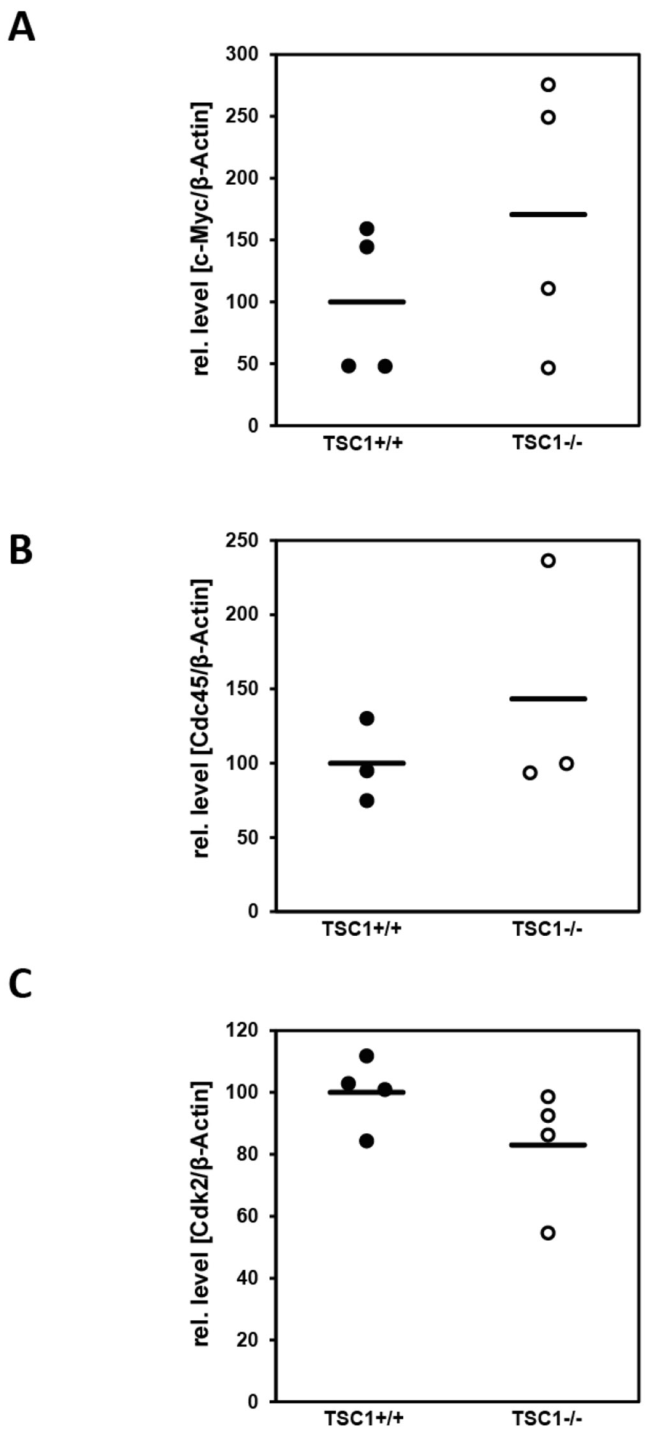

Supplementary Figure S9: S-phase regulation and DNA replication factors in TSC1$^{-/-}$ MEFs. Relative expression levels in cycling cell populations of the replication factors **A.** c-Myc, **B.** Cdc45, and **C.** the S-phase cyclin-dependent kinase Cdk2, respectively.

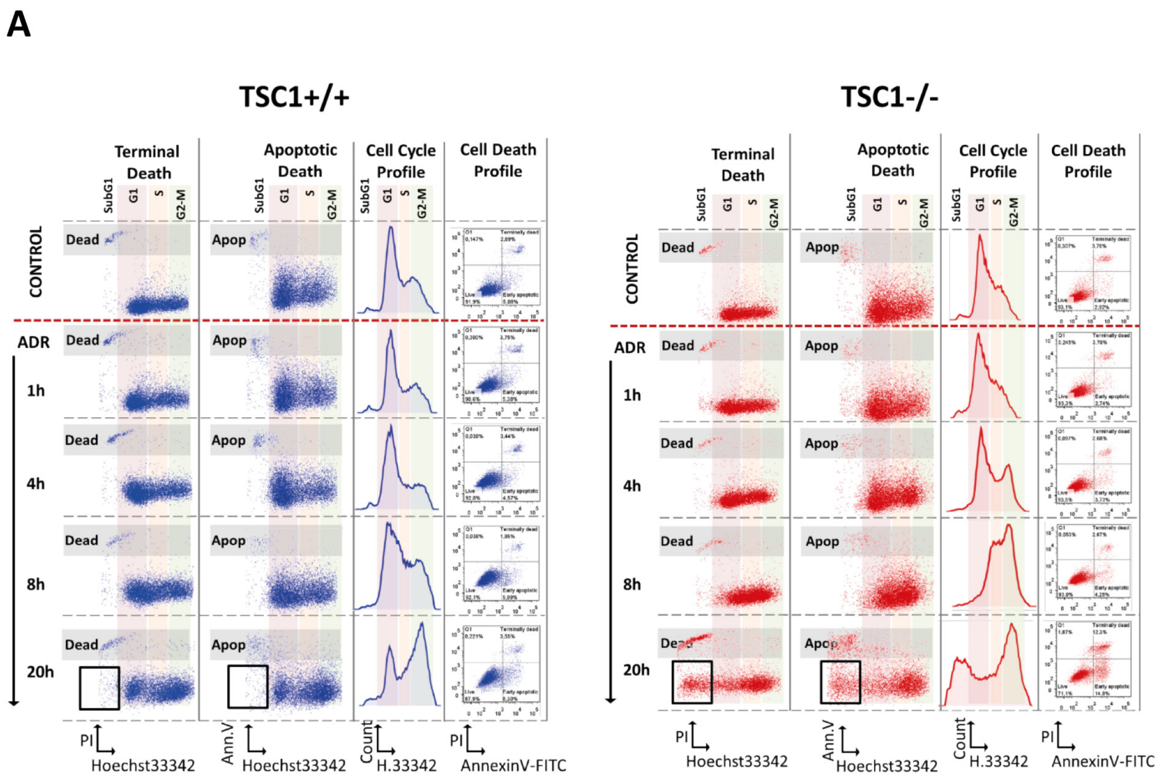

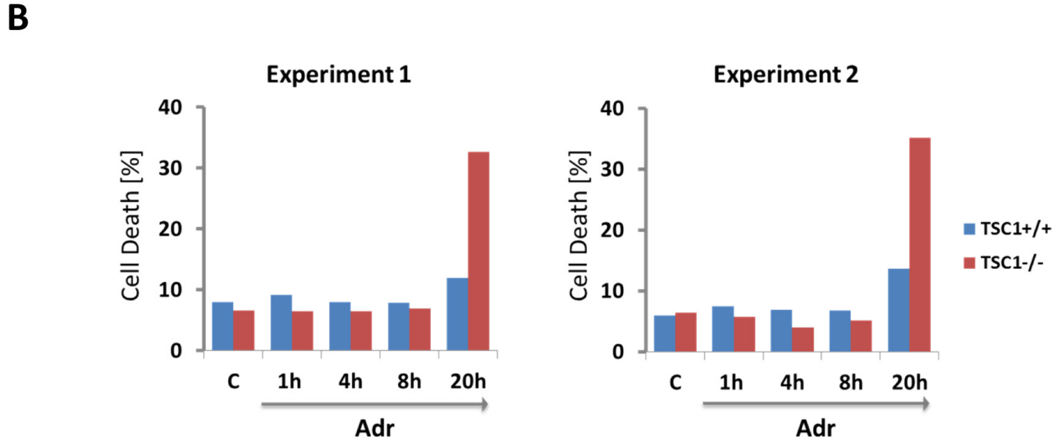

Supplementary Figure S10: Cell death kinetics following Adr treatment. A. One representative series of two experiments showing the cell cycle status (live cell DNA binding dye, Hoechst33342) in relation to either apoptosis induction (Annexin V positive) or terminal cell death (PI positive), in untreated or Adriamycin treated (0.5 µg/ml, 20 h) $TSC1^{+/+}$ and $TSC1^{-/-}$ cells. Black boxes at 20 h highlight cells with sub-G1 DNA content, but distinctly negative for both Annexin-V as well as PI in $TSC1^{-/-}$, suggestive of mitotic catastrophe. **B.** Quantification of total cell death (Annexin-V + PI positive cells) in $TSC1^{+/+}$ and $TSC1^{-/-}$ cells treated as in A. See also the right-most column in A for both cell lines. Data-sets are a mean of duplicate samples from two independent experiments. Error bars represent standard deviation (SD).

Supplementary Table S1: G2-M checkpoint analysis

Sample #	G2 Phase			Mitosis			Ratio G2/M
	Expt 1	Expt 2	Geo-Mean	Expt 1	Expt 2	Geo-Mean	
**TSC1$^{+/+}$ control**	90.2	95.1	92.62	9.81	4.89	6.93	13.37
**TSC1$^{+/+}$ 0.5h Adr**	94.7	99.7	97.17	5.35	0.292	1.25	77.74
**TSC1$^{+/+}$ 1h Adr**	98.8	99.947	99.37	1.16	0.053	0.25	400.77
**TSC1$^{+/+}$ 4h Adr**	99.7	100	99.85	0.288	0.021	0.08	1283.93
**TSC1$^{+/+}$ 8h Adr**	99.3	99.2	99.25	0.704	0.759	0.73	135.78
**TSC1$^{+/+}$ 20h Adr**	98.4	99.8	99.10	1.55	0.179	0.53	188.14
**TSC1$^{-/-}$ Control**	89.3	87.7	88.50	10.7	12.3	11.47	7.71
**TSC1$^{-/-}$ 0.5h Adr**	92.1	99.2	95.58	7.95	0.845	2.59	36.88
**TSC1$^{-/-}$ 1h Adr**	93	99.956	96.42	6.98	0.044	0.55	173.98
**TSC1$^{-/-}$ 4h Adr**	96.4	99.8	98.09	3.57	0.178	0.80	123.04
**TSC1$^{-/-}$ 8h Adr**	98.5	99.5	99.00	1.48	0.507	0.87	114.29
**TSC1$^{-/-}$ 20h Adr**	96	98.6	97.29	3.97	1.31	2.28	42.66

Table with raw data from 2 independent EdU/DAPI/pSer10-HisH3 flow cytometric cell cycle profiling for G2/M checkpoint analyses.

Supplementary Table S2: Comparison between prototypic replication stress and the `ERR´ phenotype in tuberous sclerosis cells reported here

	Feature	Untransformed (Normal) Cells	Classical Replication Stress Ras-, Myc-, Cyclin E-driven tumors	Energy Restricted Replication (ERR) phenotype
**Growth Signalling**	mTORC1 activity status	+	++ (high)	+++ (constitutive)
	p53 activity status	+	- (usually lost)	+++ (upregulated)
**Replication Properties**	Ori-Ori distance	++++	++ (drastic reduction)	+++ (marginal reduction)
	Fork Progression	++++	+++	++
	Fork Asymmetry	-	+++ (Excess stalling)	- (absent)
	Origin re-firing	-	+	-
	Nucleotide pools	++ (normal)	+ (frequently diminished)	++ (no shortage documented)
**Energetic Status**	ATP levels	+++	++ (shortage)	+ (severe shortage)
**RS Checkpoint Proficiency and Response**	ATR expression	++	++++ (frequently upregulated)	+ (downregulated)
	Chk1 expression	++	++++ (frequently upregulated)	++ (unchanged)
**Cellular outcome**	Chromosome breaks, rearrangements and instability *in vivo*	-	+++	-
	Stress sensitivity	+	- (evade apoptosis)	+++ (hypersensitive)
**Tumour Properties**	Invasive capacity and metastasis	-	+++ (malignant, aggressive)	- (Benign hamartomas)
